# Factors associated to burnout in medical residents

**DOI:** 10.1192/j.eurpsy.2026.11521

**Published:** 2026-07-31

**Authors:** F. Dhouib, O. Errabia, N. Kotti, A. Kchaou, M. Hajjaji, K. Jmal Hammami

**Affiliations:** 1 Occupational medecine Department; 2 Family Medecine Department, Faculty of medecine of Sfax, Sfax, Tunisia

## Abstract

**Introduction:**

Burnout has emerged as a significant occupational health concern in recent decades, particularly in the medical field. Healthcare professionals, especially medical residents, are among the most vulnerable groups due to the demanding nature of their training, long working hours, high workload, and the emotional intensity of patient care.

**Objectives:**

The objectives of our study were to assess burnout among medical residents and identify socio-professional risk factors for this phenomenon.

**Methods:**

This was a cross-sectional, analytical study of medical residents from September 1, 2022, to December 31, 2022, using an online questionnaire. Burnout was assessed using the Maslach Burnout Inventory.

**Results:**

During the study period, we surveyed 140 medical residents with a mean age of 27.1 ± 1.8 years. 67.8% of these participants were female and 84.4% were single. Most residents engaged in a leisure activity (79%). Regarding professional characteristics, first-year residents were the most represented (59.3%). In addition, a proportion of 41.8% of residents performed between 4 and 6 shifts per month. According to the Maslach scale, a proportion of 32.9% of residents indicated a high level of burnout. A high degree of depersonalization was noted in 34.2% of residents. A proportion of 69.8% of participants described a low degree of personal accomplishment. According to the analytical study, the risk factors for burnout in our population were: the presence of a mood disorder, working in surgical wards, and night shift work. In contrast, the protective factors for burnout were: practicing a leisure activity and working in a psychiatric ward.

**Conclusions:**

The implementation of appropriate corrective measures, such as the introduction of leisure activities or the reduction of the number of night shift, could contribute to a decrease in the prevalence of burnout among residents.

**Disclosure of Interest:**

None Declared

